# Optimization of Perovskite Gas Sensor Performance: Characterization, Measurement and Experimental Design

**DOI:** 10.3390/s17061352

**Published:** 2017-06-10

**Authors:** Francesco Bertocci, Ada Fort, Valerio Vignoli, Marco Mugnaini, Rossella Berni

**Affiliations:** 1 Department of Information Engineering and Mathematics, University of Siena, Via Roma 56, 53100 Siena, Italy; ada@diism.unisi.it (A.F.); vignoli@diism.unisi.it (V.V.); mugnaini@diism.unisi.it (M.M.); 2 Department of Statistics, Computer Science, Applications “G. Parenti”, University of Florence, Viale Morgagni 59, 50134 Florence, Italy; berni@disia.unifi.it

**Keywords:** gas sensing, carbon monoxide, electronic nose, nitrogen dioxide, split-plot design, robust process optimization

## Abstract

Eight different types of nanostructured perovskites based on YCoO${}_{3}$ with different chemical compositions are prepared as gas sensor materials, and they are studied with two target gases NO${}_{2}$ and CO. Moreover, a statistical approach is adopted to optimize their performance. The innovative contribution is carried out through a split-plot design planning and modeling, also involving random effects, for studying Metal Oxide Semiconductors (MOX) sensors in a robust design context. The statistical results prove the validity of the proposed approach; in fact, for each material type, the variation of the electrical resistance achieves a satisfactory optimized value conditional to the working temperature and by controlling for the gas concentration variability. Just to mention some results, the sensing material YCo${}_{0.9}$Pd${}_{0.1}$O${}_{3}$ (Mt1) achieved excellent solutions during the optimization procedure. In particular, Mt1 resulted in being useful and feasible for the detection of both gases, with optimal response equal to +10.23% and working temperature at $312{\;}^{\circ }$C for CO (284 ppm, from design) and response equal to −14.17% at $185{\;}^{\circ }$C for NO${}_{2}$ (16 ppm, from design). Analogously, for NO${}_{2}$ (16 ppm, from design), the material type YCo${}_{0.9}$O${}_{2.85}+1\%$Pd (Mt8) allows for optimizing the response value at −15.39% with a working temperature at $181.0{\;}^{\circ }$C, whereas for YCo${}_{0.95}$Pd${}_{0.05}$O${}_{3}$ (Mt3), the best response value is achieved at −15.40% with the temperature equal to $204{\;}^{\circ }$C.

## 1. Introduction

Perovskite metal oxides, with general formula ABO${}_{3}$, where the A-site ion is usually an alkaline earth or rare earth element and the B site ions could be 3d, 4d and 5d transition metal elements, have received great attention in the last few years, due to the their properties that make them suitable for many technological applications, such as oxygen membranes, heterogeneous catalysts, capacitors, gas sensors, etc. [1]. YCoO${}_{3}$ is a perovskite, studied by the authors, that proved to be sensitive to changes of the chemical composition of the surrounding atmosphere [2,3]. Stoichiometric, as well as defective or doped YCoO${}_{3}$ proved to be p-type semiconductors that change their conductivity as a function of oxidizing or reducing gas concentrations. This paper addresses the application of YCoO${}_{3}$-based nano-structured materials as low temperature conductometric sensors for toxic gas detection. For this kind of sensor, the working principle is based on reversible gas adsorption on the oxide surface that involves the exchange of charge between the oxide and the adsorbates, which modifies the oxide electronic conductivity either by creating depleted regions at the grain boundaries and/or by trapping/releasing free carriers. The mechanism of interaction of these materials with oxidizing and reducing gases, such as CO and NO${}_{2}$, is still under study, and further research is needed in order to gain the knowledge required for the full exploitation of their gas sensing properties.

In general, it is well known that many material characteristics and physical or chemical quantities influence the gas response [4,5]. For instance, the gas sensing behavior depends dramatically on the surface temperature, but also on the humidity and on the presence of interfering gas. As far as the material characteristics are concerned, it was shown that the bulk electronic properties, together with the bulk defect population, are relevant, but also that the surface type and the microstructure of the sensing film are very important. For example, it was pointed out that the use of a nano-structured material is usually related to a large gas response, mainly due to its large surface/volume ratio. This fact triggered a growing interest in the application of nano-structured materials for the detection of gases [6,7], and for this reason, nanoscale metal oxides, such as nanoparticles, nanospheres, nanotubes, nanowires and nanoporous materials, are routinely synthesized for the development of solid-state gas sensors with improved sensing properties [8,9]. Furthermore, the main problems of semiconductor conductometric gas sensors are the reproducibility, the stability and the reliability [10]. YCoO${}_{3}$-based perovskites are stable in long-term operation at fixed working temperature and gas concentration [11]. Therefore, the aging process of the chemical film can be limited using a low working temperature.

Concluding, the sensor response of conductometric gas sensors is a complex function of many quantities and parameters, that are only partially known. This paper proposes an approach to search the optimal working conditions of YCoO${}_{3}$ sensors by applying statistical methods, in particular the design of experiment and the Response Surface Methodology (RSM); in this direction, see also [12]. The proposed approach involves an ad hoc planning of a split-plot design as an RSM second order experimental design and the application of RS models with random effects, e.g., mixed RS models, in order to expound and to improve the contribution achieved through a previous study [13].

Since 1990s, RSM and experimental designs have played a relevant role aimed at improving some specific issues, such as modeling [14] and process optimization [15]. By considering the modeling features, many approaches are introduced in order to extend: (i) the concept of the fixed effect especially when evaluating blocks or noise variables; (ii) the relevance of the heteroscedasticity for the error variance.

Undoubtedly, when considering the sources of variabilities, the connection of RSM with the inclusion of random effects is a notable improvement in the study of those components, e.g., variance components [16], which may influence the main and operative variables of the production process [17,18]. More specifically, when considering the search for an optimal solution for the experimental variables in order to set a robust design, the inclusion of random effects within the fitted mixed response surface allows for evaluating the error components linked to noises and/or sub-experimental factors. To this end, a further improvement in this research is the application of a split-plot design [19,20], which is a notable experimental design when considering specific issues of noise variables studied through mixed effects. Moreover optimization through split-plot design has been recently involved in a Bayesian context, such as in [21]. Furthermore, we may also evaluate the context in which the experimental design is applied for optimizing products and/or process. In this direction, wireless communications and transmission are studied through a full factorial design in [22], where the received signal strength is analyzed. Similarly, in [23], an Analog to Digital Converter (ADC) channel optimization is carried out by considering random noise effects and the multi-response case; in [24], a statistical analysis is applied in order to study and to remove noises by the spectral domain. When considering the application of the well-known full factorial experimental design, as in [25], it must be noted that this design is an efficient statistical tool when fractionated and applied in an RSM context; and also, as basic design for the split-plot, as in [26]. Moreover, the application of an experimental design leads to the subsequent statistical modeling approach and, then, to the process optimization. The process optimization allows for achieving the optimal setting of factors involved in the experimental design, also including noises and the concept of robust design. In this way, process optimization could be performed by applying alternative models and algorithms, such as in [27], where the Jacobian matrix is calculated on the feasible region in order to obtain an optimal rehabilitation robot, or in [28], where the lassoregression and the TREFEXalgorithm are applied for optimizing gas sensors in an open system.

In our case-study, we applied the split-plot design and modeling for optimizing gas-sensor materials. The optimization step is carried out through an analytical approach involving an objective function based on a dual approach, in which the target (desirable) value is achieved with minimum process variability and also taking random effects into account.

Statistical results are satisfactory both considering modeling issues and optimization performance. Modeling results confirm the hypotheses assumed during the design planning, especially for gas concentration and temperatures; the optimization results are obtained by considering each target gas and the corresponding gas sensing material, conditioned to temperature range values.

This paper is organized as follows: the sensor samples and the measurement system are described in Section 2. The split-plot design is illustrated in Section 3, where the experimental planning has been detailed. The model and the optimization results are presented in Section 4. The discussion and final remarks are reported in Section 5 and Section 6, which conclude this paper.

## 2. Preparation of the Sensing Materials, Sensor Realization and Measurement System

### 2.1. Preparation of the Sensing Materials

In this paper, eight gas sensing materials are studied (see Table 1). Each material is produced as a powder; the sensing films of chemical sensors are obtained by deposition of pastes, based on the different powders. As pointed out in the Introduction, defects and doping are relevant for gas sensing properties, such as the selectivity towards a certain target gas; moreover, since the characterization of chemical sensors towards two different gases is under study, we decided to use powders with different chemical compositions, which were shown to be effective in this specific detection problem.

**Figure 1:**
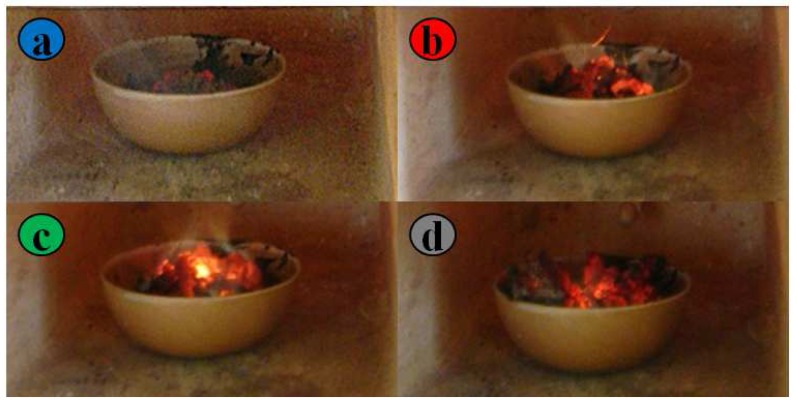
(**a**) Viscous sol into the furnace; (**b**) preliminary combustion; (**c**) propagation of flames in the sol overall volume; (**d**) end of gel combustion phase.

The materials under study were prepared by a specific route, named the gel combustion technique, that was used in order to realize nano-structured or nanoporous powders, with the aim of obtaining large surface areas and surface reactivities. In the gel combustion method, nitrate hydrates salts of Y and Co powders were mixed with citric acid and dissolved in distilled water. The solution, once heated, becomes first a sol, which then leads to the formation of a dusty grey-colored gel, which is dried at a temperature of about $100{\;}^{\circ }$C [13] and then brought to $600{\;}^{\circ }$C for 4 h. The gel bursts into flames after about ten to twenty seconds (see Figure 1) with a sudden increase of temperature, producing a spongy light black-colored powder; subsequently, the temperature is increased up to $900{\;}^{\circ }$C and maintained for 48–72 h to complete the solid state reaction. The combustion suddenly increases the temperature of the gel and exploits the instant gas expansion to produce a nanoporous grain structure, as will be shown in the images obtained by SEM analysis. Defective and doped materials are obtained by adjusting the molar ratio of the Y and Co salts and by adding Pd acetate to the mixture of nitrates of Y and Co [13,29] and, analogously, to replace a defined amount of Co/Y by an equivalent amount in moles of Pd. In this case, the Pd is expected to substitute some Y or Co atoms also in the lattice modifying also the bulk structure of the sensing materials. Alternatively, other types of Pd-doped materials were obtained by impregnation, i.e., preparing a solution of Pd acetate in acetone and adding it to the powder of YCoO${}_{3}$ in the desired proportions. The dispersion is dried in the temperature range between $500{\;}^{\circ }$C and $650{\;}^{\circ }$C for a time ranging from 6 to 18 h. The prepared powders were characterized in terms of composition and structure, using X-ray diffraction (XRD): in all cases, the predominant phase was a perovskite (>80% *w*) with a modified composition [3].

The eight materials used in this work are listed in Table 1. It can be seen that Mt5 is the stoichiometric base material, prepared with an equimolar amount of Y and Co. Mt4 and Mt6 are non-stoichiometric perovskites prepared with a Y:Co molar ratio of 1.1:1 and 1:0.9, respectively. These materials are included because they showed enhanced responses to NO${}_{2}$ [2,3].

Pd-doped materials show the best response to CO in the temperature range around (280–300) ${}^{\circ }$C [3], where the responses towards ${\mathrm{NO}}_{x}$ result in being less than 1%. Mt1 and Mt2 (YCo${}_{1-x}$Pd${}_{x}$O${}_{3}$) and Mt3 (Y${}_{1-x}$CoPd${}_{x}$O${}_{3}$) powders were prepared by substituting Co or Y with Pd. This is accomplished by adding some Pd acetate to the mixture of nitrates of Y and Co before heating [13,29]. The Co:Pd or Y:Pd molar ratio of the solution lies in the range [0.05,0.1]. Mt7 and Mt8 are obtained by impregnation, i.e., the prepared powders, YCoO${}_{3}$ and YCo${}_{0.9}$O${}_{2.85}$, are put in a solution of 1% Pd(CH${}_{3}$COO)${}_{2}$ [3]. The responses of all of the tested materials to NO${}_{x}$ are satisfactory at temperatures below 200 °C. In this range of temperature, the CO detection is negligible [3,11].

The prepared powders were characterized in terms of composition and structure by using SEM-EDAX, as discussed in the following section.

### 2.2. Morphological and Chemical Analysis of the Sensing Materials

**Figure 2:**
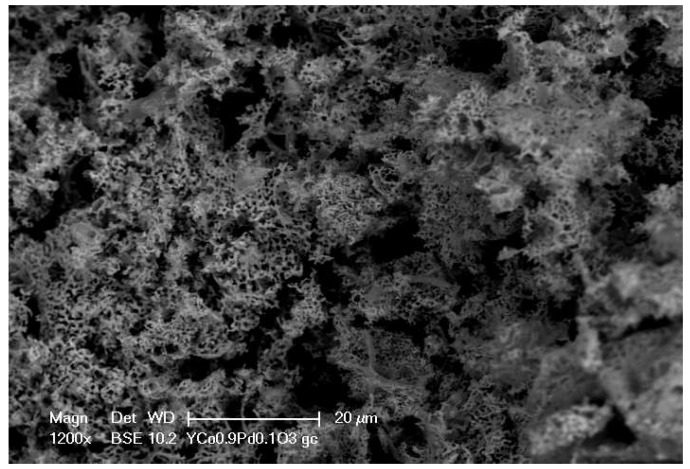
SEM: morphology analysis of Mt1. Details of spongy structure at 1200×.

**Figure 3:**
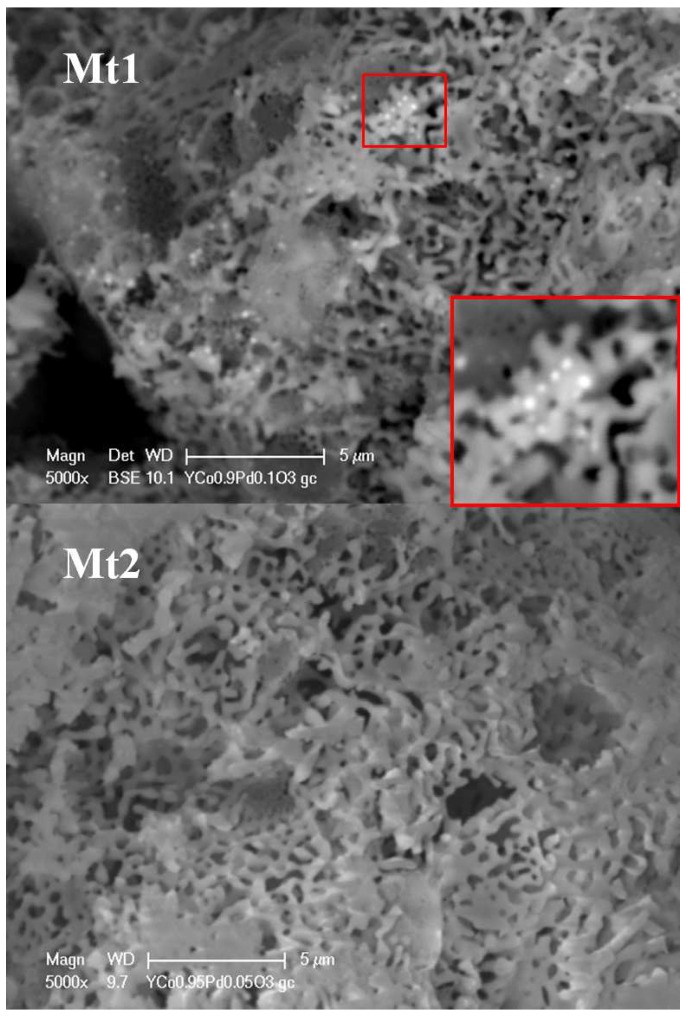
SEM: morphology analysis of Mt1 and Mt2 at 5000×.

Scanning Electron Microscope (SEM) analysis was used to gain information both on the structure of the prepared materials that, as expected, consist of nanoporous micro-grains and on their chemical composition [30,31,32,33,34,35]. The SEM image of Mt1 powder is shown in Figure 2. The spongy morphology grants a large surface area and consequently excellent characteristics as a gas sensing material. This feature can be appreciated even better at a higher magnification (Figure 3). The perovskite structure is spongy, and the distribution of nanoporous micro-grains is rather uniform. The image of Mt1 shows that palladium is also segregated on the surface as nanoparticles with diameters smaller than 50 nm. Mt2, which is doped with a lower quantity of Pd (5%w/w), does not show the presence of palladium on the surface; this suggests that, in this case, most part of the Pd is in the perovskite structure, whereas for larger quantities, some of the Pd tends to be arranged on the material surface.

**Figure 4:**
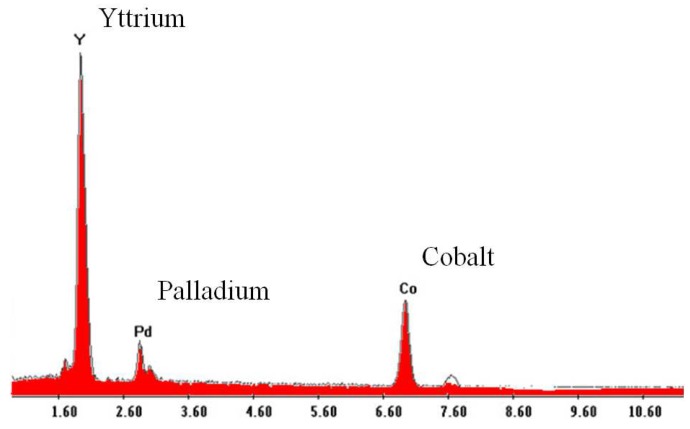
EDAX: micro-chemical analysis of Mt1.

The chemical composition of perovskites has been verified by means of Energy Dispersive X-ray Analysis (EDAX) [36]. The substitution element for Co, palladium in this case, is remarkably in compliance with the theoretical chemical formulation of 10% (Figure 4).

From the SEM and EDAX analyses, we assessed that all eight materials have been successfully synthesized by means of non time-consuming and cost-effective techniques.

### 2.3. Sensor Preparation and Measurement System Description

**Figure 5:**
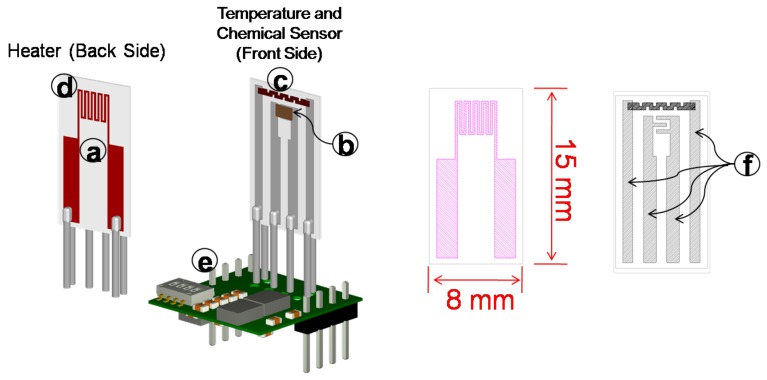
Sensor structure: (**a**) alumina substrate; (**b**) sensing film; (**c**) temperature sensor (Pt-Resistance Temperature Detector (RTD)); (**d**) heater; (**e**) conditioning and acquisition electronics; (**f**) Ag-Pt conductor material.

In order to test all of the prepared perovskites, some sensors were realized [11,37] on ad hoc alumina substrate (size 8 mm × 15 mm × 0.26 mm of thickness) (Figure 5a) equipped with electrodes for the sensing film (Figure 5b), a heater on the backside (Figure 5d) and a Pt-based Resistance Temperature Detector (RTD) (Figure 5c), all deposited by screen printing (Figure 5). The realization of RTD Pt-based sensor close to the gas sensing film allows us to obtain an accurate and consistent measurement of the operating film average temperature, which is of the utmost importance for the repeatability of the gas sensors’ characterization. The conductor material for electrodes is based on Ag/Pt, which utilizes an oxide bond system for providing particularly excellent adhesion to alumina oxide substrates.

The perovskite powders were mixed with some drops (1 mL) of dimethyl phthalate (≥99%) by obtaining a homogeneous paste, and screen printed [38], as a planar and homogeneous sensing layer across the two electrodes. After the deposition, the device is heated at $500{\;}^{\circ }$C in order to ensure the adhesion of the film to the substrate.

The variation of the electrical resistance of the so-obtained film is the sensor output. In the remaining part of this work, the sensor response is defined as follows:

(1) $$Response\left[\%\right]=\frac{\left(R-R_{0}\right)}{R_{0}}\times 100$$

In Formula (1), $R_{0}$ is the baseline resistance value, obtained at the considered temperature in a reference gas (air), whereas *R* is the resistance value after a fixed duration exposure to a target gas in a given concentration.

In this work, the experimental data were collected through the measurement system described in [11,13] developed to simultaneously characterize up to eight sensors. The sensors are placed in a circular array exploiting eight front-end boards for conditioning and acquisition electronics (Figure 5e), mounted on a main board. The system provides an accurate measurement of the gas sensing film temperature, with a resolution of $0.1{\;}^{\circ }$C, and the uncertainty of this temperature is less than $3{\;}^{\circ }$C for temperatures in the range [$120{\;}^{\circ }$C, $400{\;}^{\circ }$C].

The sensors were tested in the presence of two different toxic gases, CO and NO${}_{2}$, mixed with synthetic air (20% oxygen and 80% nitrogen) with an RH value of 0%–30% at $25{\;}^{\circ }$C. The RH value has been chosen by considering the reference based on the previous works of the authors and by preventing the condensation inside the measurement chamber.

All of the presented measurements are obtained by applying a specific protocol and by repeating each measurement three times. Data used hereafter are averaged values calculated through the three measurement results. The applied protocol consists of exposing the sensor to a constant flow of 300 mL/min; each measurement consists of 8 min in a flow of synthetic air (carrier gas), 8 min in a mixture of air and CO or NO${}_{2}$ and 8 min again in air to allow the complete recovery of the surface; so, each measurement needs 24 min. Three different concentration values ranging from 71 to 284 ppm for CO by referring to the lowest limit for the activation of consumer alarms (i.e., 70 ppm for UL 2034, Standard and multiple station carbon monoxide) and the early health effects, i.e., headache and nausea after 2–3 h of exposure over 200 ppm of CO. On the other side, we choose a range from 6 to 16 ppm of NO${}_{2}$ for contemplating a wide variety of commercial and industrial application, such as vehicle diesel exhaust in parking structures, tunnels and ventilation systems.

**Figure 6:**
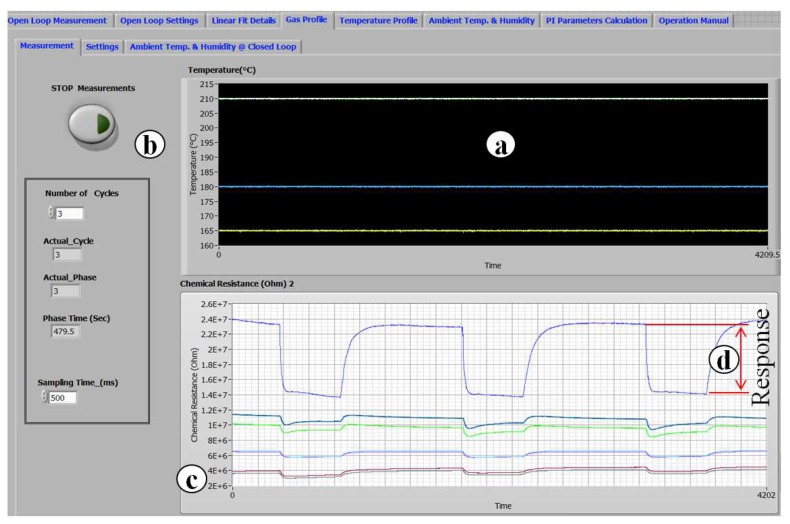
Response and temperature versus time: (**a**) working temperature versus time; (**b**) VI front panel; (**c**) chemical resistance vs. time; (**d**) amplitude of the response.

An example of the measurement data is shown in Figure 6, where seven sensor outputs during three measurement cycles are shown. In Figure 6, all of the tested materials show a satisfactory response reproducibility and a complete recovery in air after the exposition to NO${}_{2}$.

The assumption that the response of the sensors is stable over the whole measurement time has been proven in [11] for fixed values of working temperature and gas concentration in long-term operation. The responses of the material are satisfactorily stable over a long period at fixed working temperature and gas concentration values.

Furthermore, the levels of the experimental factors have been chosen by considering the quality of CO and NO${}_{2}$ detection, [11], based not only on the amplitude of the response, but also on the response time, that is the time for the sensor response signal to go from 10% to 90% of its steady state value, when the sensor response is exposed to a step change of the target gas concentration. For all of the materials under study, the response time for CO and NO${}_{2}$ is less than 2 and 4 min, respectively (i.e., around 70 s at $300{\;}^{\circ }$C in 250 ppm of CO and 230 s at $200{\;}^{\circ }$C in 10 ppm of NO${}_{2}$ for Mt1).

The other important parameter is the recovery time, which is the time it takes for the sensor signal to return to 10% of its baseline value after a concentration step change from a certain value, of the target gas, to zero. For the complete recovery in CO and NO${}_{2}$, 4 and 8 min are needed (i.e., around 180 s at $300{\;}^{\circ }$C in 250 ppm of CO and 420 s at $200{\;}^{\circ }$C in 10 ppm of NO${}_{2}$ for Mt1). However, the response and the recovery times of MOX sensors are considered one of their main challenges. Most of the MOX sensors suffer from slow speed of response and long recovery [39]. Further study and investigation can lead to the implementation of an ad hoc design of experiments devoted to minimize the response time and recovery time by taking into account the variables, i.e., temperature, concentration and type of target gas, carrier gas, flow and humidity. In what follows, the sensing film resistance, measured within the planned experimental design, was used for the modeling step, in order to evaluate linear, first order interactions and quadratic effects related to all of the variables involved in the sensor responses, as detailed in the following Section 3 and Section 4.

## 3. The Split-Plot Design

In the last two decades, split-plot design [40] has received great attention as a valid plan in the technological field and for a robust design approach, following the seminal contributions by [19,20,26,41,42]. In [19], this experimental design has been developed by considering its specific framework, in which the main role is played by the distinction between Whole-Plot (WP) and Sub-Plot (SP) factors. The bi-randomization of the split-plot design allows us to study noise and block variables as WP factors, also considering random effects; following, the experimental (main) variables, e.g., SP factors, are randomized within the WP units. Furthermore, the inclusion of experimental variables as SP factors guarantees a more accuracy estimation for these effects. The inclusion of the split-plot design as a crossed bi-randomized design implies that this design may be also considered as a second order design in a Response Surface Methodology (RSM) context [43]. Further theoretical developments contributed to expound the relevance of the split-plot design: in [20] is established the equivalence between Ordinary Least Squares (OLS) and Generalized Least Squares (GLS) estimation methods for fixed effects, if the split-plot design is planned according to specific structure.

In our case study, the split-plot design is planned for studying and optimizing the performance of the characterized gas sensing materials. We distinguish between two types of factors: the sub-experimental factors, such as block factors and noises, which are included in the whole plots; the experimental factors, e.g., process variables, which are the main object of interest and are studied as sub-plot factors.

### 3.1. Planning the Split-Plot

The step of planning for the split-plot design is started by considering all of the sources of variability involved in the process, independently from the role played by each source, e.g., experimental or sub-experimental role. For example, we distinguish between two temperatures: (i) the temperature of the measurement chamber; (ii) the temperature measured by the RTD on the sensor, also called the working temperature. The latter plays a relevant role in the final experimental design, while the first one is evaluated as an external variable.

More precisely, we planned a split-plot design with the block variable, target gas, at two-levels: two gases, NO${}_{2}$ and CO, coded as 1 and 2 in Table 2 respectively. For each target gas, three chambers are studied; eight different sensors are studied within each chamber, each sensor relating to a gas sensing material. Each chamber, within a target gas, is considered as a replicate with a specified gas concentration level. Therefore, the gas concentration is studied as a random variable, and it is included in the experimental design as a factor at three levels for each target gas, each level corresponding to a chamber. Further, a noise variable is included as the WP factor: the humidity, at two levels (wet and dry), corresponding to 0% and 35%; the humidity is induced through an a priori setting of the thermostatic bath. The gas sensing materials (eight types of perovskites) and the working temperature are considered as SP factors. The material type is then evaluated as the categorical variable at eight levels, while the temperature is studied by considering a different range of values (${}^{\circ }$C) by the target gas. In order to better evaluate the working temperature, we studied four temperature intervals for each target gas. The response variable is illustrated in Formula (1).

Therefore, for each target gas, we applied a split-plot design with two WP factors (humidity and gas concentration) and two SP factors (material type and working temperature). Each replicate is formed by a chamber obtained by a given sensors array and performed at a specific gas concentration level; within each replicate, humidity is studied at two levels, and at the SP level, the eight material types are studied according to the four levels of the working temperature. The total amount of experimental observations is 48: therefore, we have 24 observations for each target gas. The environmental temperature for each chamber has been measured during the experimentation step, and it has been involved in the statistical model as a random variable; but, it did not result in being relevant for the whole process, not even as an external variable. Other sources of variability were considered during the planning and the subsequent measurement process: (i) the difference in the heating during sensors’ fabrication, for each type of perovskite, which is included in the analysis through the material type; (ii) gas-flow, which is kept constant at 300 mL/min.

In Table 2, the detailed description of the experimental variables is reported.

### 3.2. The Split-Plot Model

Let us define the set (*Z*) of *I* whole-plot factors (noises and block) and the set (*X*) of *J* sub-plot factors. In addition, we define with WP${}_{k}$ (k=1,...,K) the combinations of levels for the *I* Whole-Plot (WP) factors; while we define with SP${}_{k}$ (k=1,...,K) the combinations of levels of the *J* Sub-Plot (SP) factors, in the replicate (block) *k*. Then, within each replicate *k*, we have $n_{k}$ = WP${}_{k}$ × SP${}_{k}$ combinations, and therefore, the total number of trials is $N=\sum_{k}^{K}n_{k}$. Furthermore, we define $z_{i}=\left(z_{1i},\;...,\;z_{ui},\;...,\;z_{ni}\right)$ as the generic vector, related to the *i*-th WP factor, (i=1,...,I); while $x_{ij}=\left(x_{1ij},\;...,\;x_{uij},\;...,\;x_{nij}\right)$ is the generic vector related to the *j*-th SP factor, (j=1,...,J). Therefore, the general second order split-plot model in an RSM setting, defined for a single observation $y_{u}$, a single replicate (K = 1) and a single response variable *Y*, is the following:

(2) $$\begin{array}{l} y_{u}\left(X,Z\right)=\beta_{0}+\sum_{i}^{I}\gamma_{i}z_{ui}+\sum_{j}^{J}\beta_{j}x_{uij}+\\ \;+\sum_{i;i<i^{\prime }}^{I-1}\sum_{i^{\prime }=i+1}^{I}\gamma_{ii^{\prime }}z_{ui}z_{ui^{\prime }}+\sum_{j;j<j^{\prime }}^{J-1}\sum_{j^{\prime }=j+1}^{J}\beta_{jj^{\prime }}x_{uij}x_{uij^{\prime }}+\\ \;+\sum_{i}^{I}\gamma_{ii}z_{ui}^{2}+\sum_{j}^{J}\beta_{jj}x_{uij}^{2}+\sum_{i}^{I}\sum_{j}^{J}\delta_{ij}z_{ui}x_{uij}+\\ \;+\psi_{u(WP_{k})}+\epsilon_{u(SP_{k})};u=1,...,n \end{array}$$

note that $\gamma_{ii^{\prime }}$ and $\beta_{jj^{\prime }}$ are the coefficients related to the interaction effects of WP and SP factors, respectively; $\gamma_{ii}$ and $\beta_{jj}$ are the coefficients of the quadratic effects of WP and SP factors, respectively; while $\delta_{ij}$ are the coefficients related to the interaction terms between WP and SP factors. These last terms play a relevant role in the robust design approach, since they contain the parameters of the control ☆ noise interaction effects. The error terms are represented by $\psi_{u(WP_{k})}$ (whole-plot error) and $\epsilon_{u(SP_{k})}$ (sub-plot error). We suppose that $\psi ∼i.i.d.\;N(0,\;\sigma_{\psi }^{2})$ and $\epsilon ∼i.i.d.\;N(0,\;\sigma_{\epsilon }^{2});$ in addition, we suppose that the error terms are uncorrelated. Note also that the first error term is formed by the interactions between the replicate effect and the sub-experimental factors; the WP effects are tested against this error component. The second error term is the residual error of the model. It must be noted that in a split-plot design, a single replicate is identified by one block. In our study, each target gas is analyzed through three replicates; each replicate is identified by a chamber and a pre-specified gas concentration level.

### 3.3. The Optimization Measure

The optimization step is carried out by applying the method explained and applied firstly in [44] and subsequently extended and improved in [45]. In the literature, many authors (among others, [46,47]) suggest different measures of optimization in order to set an optimal solution, according to the different kinds of experimental situations involved. When considering the dual response approach [14], two surfaces are estimated, one for the mean and one for the dispersion effect; in addition, weights could be introduced in the objective function; for example, in the cited literature, two global weights, one for the mean and one for dispersion, are defined. Recent and further developments could be also found in [15].

The method proposed by [44] takes the estimated model and the target value into account. In general, we may define the following distance between the estimated surface $\hat{Y}$, considered as a function of the two sets of variables, process (*X*) and noise and/or random (*Z*) variables and the target value τ:

(3) $$S\left(X,Z\right)={\left(\hat{Y}\left(X,Z\right)-\tau \right)}^{2}$$

*S* can be viewed as a crude measure of variability as a basis for the dual approach, where the adjustment to the target value is performed. The second aim is the minimization of Formula (3) on the coded experimental region:

(4) $$\min_{\left[\chi :\{X,Z\}\right]}S\left(X,Z\right)$$

It must be noted that our aim is to find the best solution for the set of factors (X,Z) by minimizing Formula (4), defined here for only one response variable, and simultaneously, to achieve the desired value for the target. In our case study, the target value for the variation of the electrical resistance of the sensor response, Formula (1), is maximized as in a larger-the-better situation also by conditioning to the working temperature.

Moreover, S(X,Z) is the surface to be optimized, and in this case, it corresponds to Model (2), estimated for each material type within a target gas, and by including fixed and random effects. The optimization measure applied in this study, even though extended to a split-plot experimental situation, is conceptually similar to the optimization measure applied in [13].

Our contribution can be summarized as follows:

1. in order to solve the problem of optimization for a split-plot design and a mixed RS model, we apply the single measure (3), which allows us to fit just one surface for the location and dispersion effects of the dependent variable [45];
2. we involve both random and fixed effects;
3. the response variable is optimized by also involving random coefficients and by including confidence interval estimates for each random variable.

Therefore, by applying Formula (3), the optimization is carried out by considering Model (2) applied within each target gas and material type and involving random and fixed effects. Each random effect (Model (2)) is included in Formula (3) through its estimated coefficient, with lower and upper bounds (confidence interval at the α=0.05 confidence level).

The relevance must be stressed of the application of the dual response approach, e.g., optimization according to mean and dispersion of the response variable, through the objective measure (4), which evaluates only one estimated RS mixed model for a split-plot design, and involving fixed, as well as random effects. The process optimization is carried out by applying the NLP procedure (SAS Software; Version 9.2, Windows Platform).

## 4. Model and Optimization Results

The statistical modeling is carried out by applying the MIXED procedure of SAS software (Version 9.2, Windows Platform) on the experimental data (N = 48) described in Section 3.1.

At the beginning, a single RS mixed model is applied for both the target gases jointly; even though the results are satisfactory, nevertheless, the gases show a different behavior by random effects, in particular error components split by replicates (chambers). However, it must be noted that the fixed part of the model, related to the main experimental and sub-experimental variables, is the same for both target gases. Therefore, in order to achieve a better optimization for each target gas and each material type, we further apply two distinct RS mixed models, one for each target gas; each applied model differentiates by considering the random part.

All of the experimental quantitative variables are standardized; nevertheless, the optimization results are reported in the original scale.

### 4.1. Model Results on NO${}_{2}$

A mixed response surface model is estimated for NO${}_{2}$ by taking into account the sensing films all together, also named the material type (Mtx). Here, we have 24 experimental observations, e.g., three chambers (replicates), each chamber containing eight sensing films. More precisely, the fixed part of the model involves: the sensing films as categorical variables at eight levels (see Table 1), the working temperature, in the linear and quadratic effects, and the first order interaction between the temperature and the sensing films. The random effects, also included in the mixed response surface model, are the gas concentration and the first order interaction between temperature and the three chambers. Therefore, four random coefficients are estimated. It must be noted that for this target gas, NO${}_{2}$, humidity and environmental temperature are not relevant in the final model. Furthermore, the estimation of the fixed part of Model (5) is conducted via the consolidated estimation method of Generalized Least Squares (GLS), which enlarges the Ordinary Least Squares (OLS) method in order to consider the variance-covariance matrix for the response variable [16]; moreover, the denominator degrees of freedom are calculated according to the Kenward–Roger method, which works well for the small sample size [18]. Variance components are estimated through the REML-Restricted Maximum Likelihood estimation method, which performs residual (restricted) maximum likelihood; for details, see also [16].

The specific mixed RS model, estimated for NO${}_{2}$, is the following:

(5) $$\begin{array}{c} y_{u}\left(x,z\right)=\beta_{0}+\beta_{T}\times x_{u_{T}}+\beta_{TT}\times x_{u_{T}}^{2}\\ +\sum_{i}^{8}\beta_{i}\times x_{u_{Mt_{i}}}+\sum_{i=1}^{8}\beta_{iT}\times x_{u_{Mt_{i}}}\times x_{u_{T}}+\\ +\gamma_{Gc}\times z_{u_{Gc}}+\sum_{j=1}^{3}\gamma_{K_{j}}\times x_{u_{T}}\times z_{u_{K_{j}}}\\ \left(u=1,\;...,\;24\right) \end{array}$$

In Formula (5), $\beta_{T}$ is the coefficient for the linear effect of temperature, while $\beta_{TT}$ is related to the estimation of the quadratic effect for this SP factor; $\beta_{i};\;i=1,\;...,\;8$ are the coefficients related to the material type, which is a categorical variable at eight levels and denoted here as $Mt_{i}$ for the generic *i* level; $\beta_{iT}$ are the eight estimated coefficients relating to the interaction term between these two fixed effects. It must be noted that for the categorical variable material type and for the interaction effect, the highest level is used as reference level in model estimation, i.e., $\beta_{8}$ and $\beta_{8T}$, respectively. By considering the random effects, $\gamma_{K_{j}}$ (j=1,...,3) are the three coefficients related to the evaluation of the interaction between the three chambers and the working temperature. The other random component of the model is the gas concentration $\gamma_{Gc}$.

In Table 3, the estimated coefficients for fixed effects of Model (5) are shown; it must be noted that the linear effect of material type is also relevant for setting the working temperature, through the first order interaction effect. Temperature is very relevant as a linear effect, as well as a quadratic effect. Furthermore, the first order interaction between material type and temperature plays a very relevant role for the subsequent optimization process (Section 4.3).

Table 4 shows the REML estimates related to the random effects included in Model (5).

Furthermore, we also illustrate the fixed effects by the amount of variability explained, through the Type III test, as shown in Table 5. The F-test explains the global relevance for each fixed effect involved in Model (5); it must be noted that the main effect of material type is globally highly significant; while the interaction between material type and temperature is nearly significant at 10%.

### 4.2. Model Results on CO

Analogously to the mixed response surface model estimated for NO${}_{2}$, in this subsection, we show the modeling results related to CO, by taking the sensing films (Mtx) all together into account. In this case, the 24 experimental observations, related to the three chambers (replicates), are also used to better evaluate the error variance component; in fact, the error is split according to each chamber. By considering the fixed part of the model, we evaluate: the sensing films as categorical variables at eight levels (see Table 1), the working temperature, in the linear and quadratic effects, and the first order interaction between the temperature and the sensing films. For this type of gas, the WP factor humidity is relevant for a better fitting of the model, and thus, it is included as a fixed effect. The random part of the model differentiates with respect to Model (5) in the evaluation of the error component, while only one random effect is included: the gas concentration. Therefore, we estimate a single random coefficient for gas concentration and four variance components, related to the gas concentration and the three components of the error variance split by the three chambers. It must be noted that also for the target gas CO, the environmental temperature is not relevant in the final selected model.

The specific mixed RS model, estimated for CO, is the following:

(6) $$\begin{array}{c} y_{u}\left(x,z\right)=\beta_{0}+\beta_{T}\times x_{u_{T}}+\beta_{TT}\times x_{u_{T}}^{2}\\ +\sum_{i}^{8}\beta_{i}\times x_{u_{Mt_{i}}}+\sum_{i=1}^{8}\beta_{iT}\times x_{u_{Mt_{i}}}\times x_{u_{T}}+\beta_{H}\times x_{u_{H}}\\ +\gamma_{Gc}\times z_{u_{Gc}}+\sum_{j=1}^{3}\gamma_{\epsilon_{\left(K_{j}\right)}}\\ \left(u=1,\;...,\;24\right) \end{array}$$

In Formula (6), $\beta_{T}$ is the coefficient for the linear effect of temperature, while $\beta_{TT}$ is related to the estimation of the quadratic effect for this SP factor; $\beta_{i};\;i=1,\;...,\;8$ are the coefficients related to the material type, which is a categorical variable at eight levels and denoted here as $Mt_{i}$ for the generic *i* level; $\beta_{iT}$ are the eight estimated coefficients relating to the interaction term between these two fixed effects. A further fixed coefficient $\beta_{H}$ is related to the linear effect of the WP factor humidity.

By considering the random part of the model, $\gamma_{\epsilon_{\left(K_{j}\right)}};\;\left(j\;=\;1,\;...,\;3\right)$ are the coefficients related to the evaluation of random error component split by (K=3) chambers. The random effect of the model is the gas concentration $\gamma_{Gc}$.

In Table 6, the estimated coefficients for the fixed effects of Model (6) are shown; it must be noted that the linear effect of material type is more relevant for this type of gas; the working temperature is relevant both in the linear and quadratic effects. Furthermore, the first order interaction between material type and temperature shows a better fitting through the estimated coefficients, also due to the standard errors, which are lower than the corresponding standard errors estimated in Model (5) (Table 3).

Table 7 shows the REML estimates related to the variance components included in Model (6).

In Table 8, the fixed effects by the amount of variability explained, through the Type III test, are reported. The F-values explain the global relevance for each fixed effect involved in Model (6); the highly significant p-value must be noted for the material type effect and also for the linear effect of the working temperature. The interaction between temperature and material type is also significant (α=5%), while the quadratic effect of the temperature shows a low importance.

### 4.3. Optimization Results

The process optimization is carried out by applying Formulas (3) and (4) reported in Section 3.3. Moreover, the two estimated models, one for each target gas (Formulas (5) and (6)), are then used to compute Formula (3), by considering the specific features for this process and the peculiar characteristics of the experimental variables involved therein. Therefore, during the optimization step, a further distinction is performed by considering the type of effect, fixed or random, and the nature of variables. All of the procedures are carried out on the coded experimental region, even though the results are reported on the original scale.

Furthermore, in the case study, the target value τ for the sensor response values (%) is maximized for each material type within the target gas, and simultaneously, a minimum for the working temperature has been investigated, within constrained and specified intervals, as shown in Table 2, e.g., $\left[165,210\right]{\;}^{\circ }$C and $\left[240,310\right]{\;}^{\circ }$C for NO${}_{2}$ and CO, respectively. The work in [11] shows that the aging process of the chemical film can be limited using a low working temperature by providing savings in terms of electrical power consumption. The achieved optimal solution, related to the maximization of the sensor response, may be viewed as a global performance measure for each material type, by considering the related gas concentration, the working temperature and the other effects involved in the models, also considering first order interactions.

Note that each sensor film is optimized separately within a target gas, through the estimated RS model, because the eight sensing films are included in the split-plot design as a categorical variable at eight levels. For each film, the variability of the estimated coefficient is evaluated by including the confidence interval (lower and upper bounds) in the optimization code. The other fixed effect of the model, temperature, is also evaluated through the estimated coefficient and the range on the coded experimental region. When considering the interaction between sensor film and temperature, this effect is involved in the optimization step by the estimated coefficients, one for each material type, but also including a control on coefficient variability through the related confidence interval. Random effects, e.g., gas concentration, the related first order interaction (Model (5)) and error components (Model (6)) are included in the objective function S(X,Z) by confidence intervals.

The optimal solutions are reported at the original level, for each target gas (Table 9 and Table 10); more precisely, for NO${}_{2}$ (Table 9), we illustrate the achieved results for material type Nos. 1, 3, 8; for CO (Table 10), we report the results obtained for material type Nos. 1, 5. When observing Table 9, for each material type (Nos. 1, 3, 8), the achieved optimal response value (τ) and the working temperature (temp) are shown. Moreover, these results are reached by also involving the gas concentration as a random effect (−1.6551) through the REML estimation (Table 4) and the corresponding lower and upper values of the confidence interval. In addition, the interaction between each chamber and the working temperature (Table 4) is evaluated, and these three variance components are useful for a better achievement of the optimal solutions, even though they are not significant. As regards the second target gas CO, in Table 10, optimization results are reported for the material types No. 1 and No. 5. Analogously, optimal achieved values (τ) for the response conditional to the working temperatures are reported for both materials. In this case, the REML estimate (1.8869) for gas concentration is reported in Table 7, and it is also included in the optimization step with the corresponding lower and upper limits (confidence interval). It must be noted that for CO, the variance components for the residual error are split by the three chambers, in order to better evaluate the variability of each replicate; these variance components are also included in the optimization step for better controlling the achievement of the optimal response conditional to the temperature values.

Optimization results are checked through the objective function value (of), the gradient estimates (maximum absolute gradient value) $\left(∥x{∥}_{\infty }\right)$ and the determinant of the Hessian matrix (|H|). A last remark relates to the attention paid to avoid constrained and unstable optimal solutions; a further check is the specified limit equal to an upper-bound for the step length (0.1) of the line search algorithm, during the first *n* iterations.

## 5. Discussion

The designed and implemented MOX chemical sensors appear complex; therefore, this research allowed us to identify the main effects and their associations among the experimental and sub-experimental factors involved in the planned split-plot design, by also including the related variability, in order to assess the response characteristics of perovskite materials under CO and NO${}_{2}$ atmospheres. The applied split-plot design displayed an excellent performance for searching the optimum point of the response conditionally to the working temperature. The obtained response measurements for the eight analyzed materials appear comparable with the well-known YCO${}_{3}$ background [2,37]. Furthermore, the experimental design is planned by considering a very small amount of trials (*N* = 48), 24 observations for each target gas. The experimental data are then modeled through mixed response surface models for taking into account fixed, as well as random effects, specifically noises, e.g., humidity, and replicates, identified by chambers (three ones for each target gas); each chamber settled at a specific level of gas concentration. The choice of planning a split-plot design is related to the peculiar characteristics of this design when considering the distinction between WP factors and SP factors. The process optimization is carried out by involving: (i) the specific design variables, e.g., experimental and sub-experimental; (ii) the role played by each variable in this study. By considering the obtained optimization results, we make a distinction between the two target gases.

As for TG.1—Target Gas.1 (Table 9), e.g., NO${}_{2}$ (16 ppm, from design), Mt1 achieved several acceptable solutions during the optimization step; however, the best final solution is reached for the response value at −14.17%. Similarly, the material type Mt8, YCo${}_{0.9}$O${}_{2.85}+1\%$Pd, achieved the best solution with the response value optimized at −15.39% with the working temperature at $181.0{\;}^{\circ }$C, whereas for Mt3, YCo${}_{0.95}$Pd${}_{0.05}$O${}_{3}$, the best achieved response value is equal to −15.40% with temperature equal to $204{\;}^{\circ }$C. Unfortunately, the other material types gave less acceptable solutions from the engineering point of view, even though they satisfied all of the statistical requirements.

As regards TG.2 (Table 10), e.g., CO, better results are obtained from the statistical point of view, especially by considering all of the diagnostic measures (convergence criteria and objective function values). Excellent results are achieved during the optimization procedure for Mt1. In particular, YCo${}_{0.9}$Pd${}_{0.1}$O${}_{3}$ is useful and feasible for the detection of both gases, with optimal response equal to +10.23% and working temperature at $312{\;}^{\circ }$C for CO (284 ppm, from design) and response equal to −14.17% at $185{\;}^{\circ }$C for NO${}_{2}$ (16 ppm, from design). In this context, the second sensing film, Mt5, YCoO${}_{3}$, is optimized at a good response value equal to +9.86% with temperature equal to $309{\;}^{\circ }$C. For this specific gas, CO, the other six sensing films show good performances from the statistical point of view; nevertheless, the engineering interpretation requires further studies.

When considering the gas concentration, the estimated random effects, reported in Table 4 and Table 7, allow for setting the optimization results for this variable into a range of variability equal to [16, 21 ppm] for TG.1 and equal to [15, 17.1 ppm] for TG.2.

The statistical study here applied reinforces the technical aims, also for providing the optimization of gas sensing materials conditioning to temperatures.

**Figure 7:**
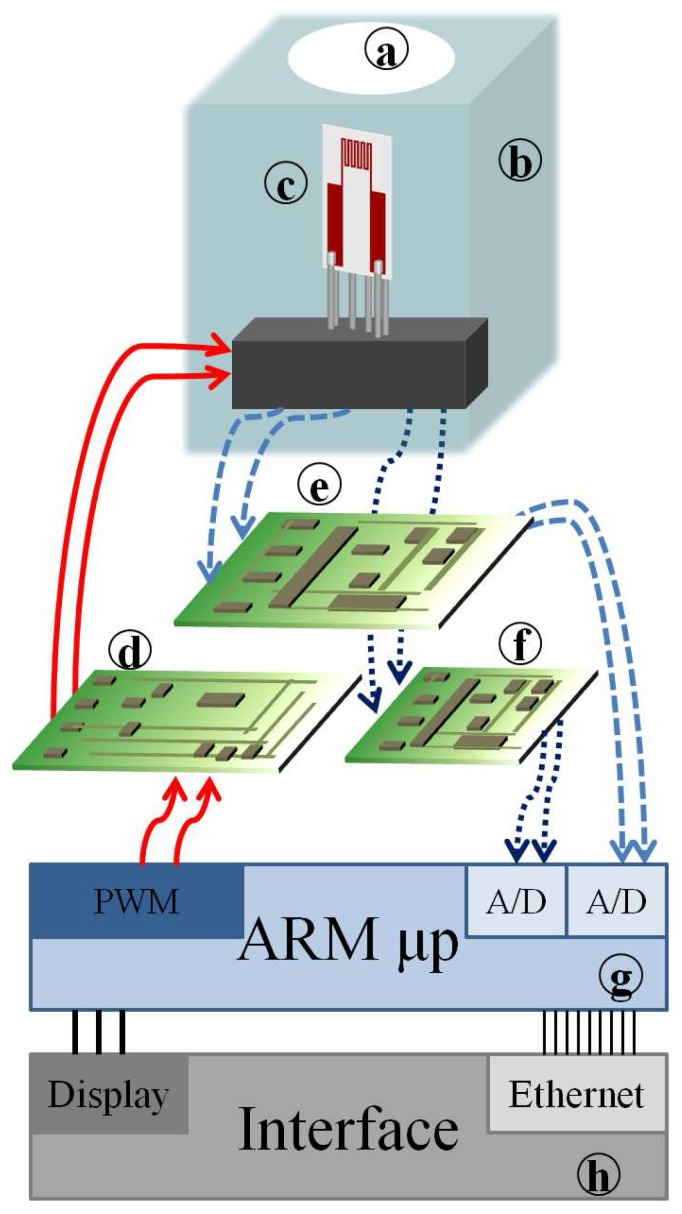
Portable system structure for industrial use. (**a**) Opening; (**b**) enclosure of chemical sampling; (**c**) sensor; (**d**) eater driver; (**e**) R-Vconverter (large range); (**f**) R-V converter (low range); (**g**) μ-processor ARM; (**h**) interface.

In this context, the proposed materials can be used for on-site detection of combustion of by-products (CO and NO${}_{2}$) in industrial applications where a complete detection system is based on the architecture shown in Figure 7. This system allows for programming the operating temperatures of the sensors and hence for optimizing the response to the target gases both for CO and NO${}_{2}$.

The portable system (Figure 7) is based on an ad hoc substrate (c) hosting both the sensing film (i.e., Mt1, Mt2, Mt5 and Mt8), the heater and RTD temperature sensors, and it allows for implementing a simple, fast and accurate working temperature control system. The front-end is designed in order to grant a high accuracy of temperature measurement and control (e) and a very large measurement range for the resistance of chemical film (f). Moreover, the overall design of the sensor aims at reducing the power consumption (d). A low-cost microprocessor, ARM (g), with a Reduced Instruction Set Computing (RISC) architecture, controls the system, acquires and processes the measurement data. This architecture provides all of the requirements for stand-alone devices. An Ethernet interface allows for easy connectivity (h). By regarding the contribution of this work in conjunction with the previous study [13], a significant improvement in the detection of dangerous gases has been provided. Moreover, the significant improvement can also be acknowledged by considering the methodological approach, through the planning and optimization of the split-plot with the inclusion of environmental noise effects, such as humidity.

## 6. Final Remarks

This work dealt with the optimization of the metal oxide semiconductor based on gas sensors using a novel class of materials. The new approach, based on statistical planning and evaluation of characterization measurements, led to an identification of optimal working temperature (for high sensitivities) without having to do a large size of experimental trials.

Perovskites based on YCoO${}_{3}$ have been developed for the detection of harmful gas and for the industrial applications; research on these materials that aims at studying and at characterizing gas sensitivity and selectivity, together with their stability and the reliability, is on-going. In particular, YCoO${}_{3}$ materials have a great potentiality to replace gas sensors with high working temperature in the electronics industry. To this end, this research is a further development with respect to a previous statistical analysis on the assessment and optimization of sensor performance by mixed response surface models. The improvement consists of the innovative contribution of a planned experimental design and the obtained improved results. In addition, the small size of the experimental data must be underlined, which can be obtained in a reasonable time and which were used to explore the complex behavior of the proposed sensors. In order to continue the research activity on the detection of YCoO${}_{3}$ in a toxic gases environment, characterizations and measurement in H${}_{2}$S and NH${}_{3}$ will be planned and implemented in an inert environment (nitrogen) and in the presence of oxygen (air).

## Figures and Tables

**Table 1:** Gas sensing materials: Chemical composition, symbols and heat-treatment.

Chemical Composition	Symbol	Heat-Treatment
YCo${}_{0.9}$Pd${}_{0.1}$O${}_{3}$	Mt1	Furnace
YCo${}_{0.95}$Pd${}_{0.05}$O${}_{3}$	Mt2	Furnace
Y${}_{0.95}$CoPd${}_{0.05}$O${}_{3}$	Mt3	Furnace
Y${}_{1.1}$CoO${}_{3.15}$	Mt4	Furnace
YCoO${}_{3}$	Mt5	Furnace
YCo${}_{0.9}$O${}_{2.85}$	Mt6	Furnace
YCoO${}_{3}+1\%$Pd	Mt7	Furnace
YCo${}_{0.9}$O${}_{2.85}+1\%$Pd	Mt8	Furnace

**Table 2:** Experimental factors. WP, Whole-Plot; SP, Sub-Plot.

Type of Factor	Name	Symbol	Levels	Blocks (Target Gas)
WP	humidity	$x_{H}$	0%;35%	1, 2
WP	gas concentration	$z_{Gc}$	6.33;11.09;15.83	1
WP	gas concentration	$z_{Gc}$	71.00;142.00;284.00	2
SP	material type	$x_{Mt_{i}}$	see Table 1	1, 2
SP	working temp.	$x_{T}$	165;180;195;210	1
SP	working temp.	$x_{T}$	240;265;285;310	2

**Table 3:** GLS estimates for fixed effects of Model (5) related to NO${}_{2}$.

Coefficient	Estimate	Standard Error	*p*-Value
$\beta_{0}$	−14.2909	1.9202	0.0017
$\beta_{T}$	9.5295	3.7842	0.1281
$\beta_{TT}$	5.1928	1.3056	0.0578
$\beta_{1}$	3.7789	3.1324	0.2941
$\beta_{2}$	13.3477	4.8880	0.0524
$\beta_{3}$	−0.8026	2.7963	0.7883
$\beta_{4}$	−25.7882	3.3729	0.0016
$\beta_{5}$	−24.5406	2.8263	0.0010
$\beta_{6}$	−25.2763	2.7555	0.0008
$\beta_{7}$	−10.2533	2.8375	0.0225
$\beta_{8}$	0	.	.
$\beta_{1T}$	−9.6419	3.5326	0.0525
$\beta_{2T}$	6.5926	5.7192	0.3132
$\beta_{3T}$	−5.1460	3.2884	0.1927
$\beta_{4T}$	2.1771	2.9047	0.4952
$\beta_{5T}$	−1.2592	4.0813	0.7731
$\beta_{6T}$	3.2455	3.7734	0.4382
$\beta_{7T}$	−2.0915	3.9481	0.6243
$\beta_{8T}$	0	.	.

**Table 4:** REML estimates for random effects of Model (5) related to NO${}_{2}$.

Effect	REML Estimate	s.e.	t-Value	*p*-Value
Gas Concentration	−1.6551	0.8639	−1.92	0.1279
Temp × chamber♯1	5.1300	3.0597	1.68	0.1689
Temp × chamber♯2	−3.0091	3.0740	−0.98	0.3831
Temp × chamber♯3	−2.1209	3.1096	−0.68	0.5327

**Table 5:** Type III test of fixed effects of Model (5) related to NO${}_{2}$.

Effect	Numerator-Degree of Freedom	Denominator-Degree of Freedom	F-Value	*p*-Value
*Mt*	7	4	38.92	0.0016
Temp	1	2	9.11	0.0944
Temp${}^{2}$	1	2	15.82	0.0578
Temp × *Mt*	7	4	3.80	0.1072

**Table 6:** GLS estimates for fixed effects of Model (6) related to CO.

Coefficient	Estimate	s.e.	*p*-Value
$\beta_{0}$	9.1466	0.8784	0.0001
$\beta_{T}$	−1.8529	0.6575	0.0390
$\beta_{TT}$	−0.7926	0.4401	0.1316
$\beta_{1}$	−3.3777	0.7757	0.0073
$\beta_{2}$	−0.2448	0.8413	0.7827
$\beta_{3}$	1.7790	0.9067	0.1070
$\beta_{4}$	−4.8633	0.9833	0.0043
$\beta_{5}$	3.1889	0.7982	0.0104
$\beta_{6}$	−3.5167	1.2448	0.0369
$\beta_{7}$	7.8001	0.9431	0.0004
$\beta_{8}$	0	.	.
$\beta_{1T}$	1.9565	0.6361	0.0276
$\beta_{2T}$	0.9972	0.9903	0.3602
$\beta_{3T}$	0.6597	1.0598	0.5609
$\beta_{4T}$	1.6621	1.0951	0.1895
$\beta_{5T}$	−3.3339	1.1847	0.0374
$\beta_{6T}$	−1.1916	1.6214	0.4954
$\beta_{7T}$	−2.1107	1.4093	0.1945
$\beta_{8T}$	0	.	.
$\beta_{H}$	0.1605	0.3930	0.6999

**Table 7:** REML estimates and variance components for the random part of Model (6) related to CO.

Effect	REML Estimate	s.e.	*p*-Value
Gas Concentration	1.8869	0.3519	0.0030
Error component			
chamber♯1	1.1818	3.3940	0.3638
chamber♯2	3.8948	3.5724	0.1378
chamber♯3	$1.39\times 10^{-8}$	$0.5\times 10^{-4}$	0.4999

**Table 8:** Type III test of fixed effects of Model (6) related to CO.

Effect	num-df	den-df	F-Value	*p*-Value
Mt	7	5	106.03	<0.0000
Temp	1	5	52.77	0.0008
Temp${}^{2}$	1	5	3.24	0.1316
Temp × Mt	7	5	7.18	0.0227
Humidity	1	5	0.17	0.6999

**Table 9:** Optimization results for NO${}_{2}$ and three material types Mti;i=1,3,8.

Results	Mt1	Mt3	Mt8
τ	−14.17%	−15.40%	−15.39%
Temp.	185.32$\;{\;}^{\circ }$C	204.0$\;{\;}^{\circ }$C	181.0$\;{\;}^{\circ }$C
*of*	$<1.8\times 10^{-10}$	$<6.1\times 10^{-10}$	$<2.3\times 10^{-15}$
$\left(∥x{∥}_{\infty }\right)$	$8.6\times 10^{-9}$	$2.7\times 10^{-9}$	$6.4\times 10^{-15}$
|H|	$<1.0\times 10^{-10}$	$<1.0\times 10^{-10}$	$<1.0\times 10^{-10}$

**Table 10:** Optimization results for CO and two material types Mt*i*; i=1,5.

Results	Mt1	Mt5
τ	10.23%	9.86%
Temp.	312.7$\;{\;}^{\circ }$C	309.8$\;{\;}^{\circ }$C
*of*	$<5.7\times 10^{-15}$	$<4.3\times 10^{-10}$
$\left(∥x{∥}_{\infty }\right)$	$7.9\times 10^{-16}$	$8.4\times 10^{-16}$
|H|	$<1.0\times 10^{-10}$	$<1.0\times 10^{-10}$
